# Short-Term FIFA 11+ Improves Agility and Jump Performance in Young Soccer Players

**DOI:** 10.3390/ijerph17062017

**Published:** 2020-03-18

**Authors:** Nebojša Trajković, Marko Gušić, Slavko Molnar, Draženka Mačak, Dejan M. Madić, Špela Bogataj

**Affiliations:** 1 Faculty of Sport and Physical Education, University of Novi Sad, Novi Sad 21000, Serbia; nele_trajce@yahoo.com (N.T.); gusicmarko@yahoo.com (M.G.); molslavko@gmail.com (S.M.); macak.md@yahoo.com (D.M.); dekimadic@gmail.com (D.M.M.); 2 Faculty of Sport, University of Ljubljana, Ljubljana 1000, Slovenia; 3 Department of Nephrology, University Medical Centre, Ljubljana 1000, Slovenia

**Keywords:** FIFA 11+, injury prevention, soccer, explosive power

## Abstract

Studies dealing with the effectiveness of the Fédération Internationale de Football Association (FIFA) 11+ prevention program to improve performance outcomes in children aged < 14 years are limited. This study aimed to point out the effects of the application of short-term FIFA 11+ warm-up program on physical performance in young football players. Participants were 36 youth male football players, divided into a FIFA 11+ (n = 19; mean (SD) age: 11.15 (0.79) y) and a control group (CG: n = 17; age: 10.87 (0.8) y) and trained for 4 weeks. Before and after the training period, standing long jump performance, agility, repeated sprint ability, sit and reach, and “30–15” intermittent fitness tests were assessed. A mixed ANOVA showed significant differences between the groups in the standing long jump test (FIFA 11+: 5.6% vs. CG: −1.9%) in favor of FIFA 11+ over CG. Additionally, the FIFA 11+ performance of the Illinois agility test was significantly better compared to the CG performance (FIFA 11+: −1.9% vs. CG: 0.03%). The main findings of this study suggest that just 4 weeks of implementation of the FIFA 11+ improves physical performance compared with traditional warm-up routines in young soccer players.

## 1. Introduction

Fédération Internationale de Football Association (FIFA) 11+ is a complete, simple, and easy to implement warm-up program, comprised of 10 conditioning exercises. The application of the FIFA 11+ program has been primarily focused on preventing and lowering incidence of injuries in soccer players [1,2], but it also may improve physical fitness to some extent [3]. Recent meta-analysis confirmed that FIFA 11+ can be considered as a fundamental tool to minimize the risks of participation in football [4]. Furthermore, along with demonstrated injury prevention, improvements in physical performance were also documented in football following FIFA 11+ program implementation [5]. The FIFA 11+ program can enhance the lower extremity balance [6], core stability [7], knee strength [7,8,9], and sprinting and jumping ability [7,10,11] in soccer players.

Zarei et al. have shown superior results of 7.5% in vertical jump height, 2.6% in Illinois agility test in favor of the FIFA 11+ program compared to standard warming up, and possible positive effects have been found in 9.1 m sprint test of −3.1% [11]. In another study, Akbari et al. found positive effects of the FIFA 11+ program on vertical jump in young football players [12]. The same study showed that 1 month after the FIFA 11+ warm up was abandoned there were no longer statistically significant differences found. Therefore, the FIFA 11+ program should be included in practice as warming up during the whole season. The long-term effects of the FIFA 11+ program were confirmed by Zarei et al. where superior results were found in favor of the FIFA 11+ program over standard warming up in vertical jump height, Illinois agility test, and possible positive effects in 9.1 m sprint test after 30 weeks [11]. 

Chen et al. aimed to determine acute effects of the FIFA 11+ program on physical performance [13]. They concluded that the effects of FIFA 11+ immediately after its application slightly reduced performance, but the performance was improved following the rest and the effects lasted 30 min after the application of the FIFA 11+ warm-up.

Furthermore, it has been shown that young boys (mean ± SD: age 10.4 ± 1.4) improved countermovement jump and 20 m sprint by 6% and 2%, respectively, following the 6-week-FIFA 11 warm-up program [14]. In 11-year-old boys, Pomares-Noguera et al. found that the FIFA 11+ kids warm-up program had possibly beneficial effects (mean difference) on the standing long jump height (3.1 cm), the performance of the Y balance test (directions: anterior (1.4 cm), posteromedial (3.5 cm), and posterolateral (3.1 cm)) and the Illinois agility test (−0.41 s), but very likely and likely beneficial effects on the countermovement jump height (1.8 cm), and the drop jump height (1.8 cm), respectively [15].

Although the main purpose of FIFA 11+ is injury prevention [1,4,11], the knowledge of training effects elicited by these training program on physical performance can help in identifying the potential mechanisms behind the reported reduction in injury incidence. Notwithstanding that studies have shown positive training effects of FIFA 11+ on measures of physical performance, only some authors have examined the exercise effects on various physical performance variables in young football players [3,5,7].

Therefore, an evaluation of the effects of the original 11+ program on football athletes younger than 14 years of age is warranted. This study aimed to point out the effects of the application of short-term FIFA 11+ warm-up program on physical performance in young football players. We hypothesized that the FIFA 11+ program would show better effects on physical performance in comparison to the traditional warm up in football. 

## 2. Materials and Methods 

### 2.1. Sample of Subjects

The sample included 36 youth male football players, aged 10–12 years, and was divided into two groups (FIFA 11+ or control group). The FIFA 11+ group consisted of 19 football players (age: 11.15 ± 0.79 years; sports experience: 3.26 ± 1.66; body height: 148.21 ± 8.58 cm; body weight: 41.55 ± 9.36 kg) and the control group consisted of 17 football players (age: 10.87 ± 0.8; sports experience: 3.42 ± 1.22; body height: 150.84 ± 6.59 cm; body weight: 42.66 ± 7.2 kg). Subjects were recruited from two different soccer teams with similar competitive schedule (one competitive game per week) and soccer drills used during their weekly training sessions. There were four inclusion criteria: (1) subjects did not have history of injury or musculoskeletal disorder in the past 6 months prior to the study, (2) aged from 10 to 12 years, (3) with at least 1 year of soccer training experience, and (4) participated in soccer training session three times per week. In addition, the exclusion criteria were as follows: missed two consecutive training sessions and missed one testing session. The study was carried out following the Declaration of Helsinki and the experimental treatment was approved by the Institutional review board of Faculty of Sport and Physical Education, University of Novi Sad (Ref. No.31/2018; approved in October, 2018). The parents provided their written consent and agreed that their children can participate in this study. 

### 2.2. Testing Procedures

The initial and final measurements were carried out in March 2019 and April 2019, respectively. Subjects followed a familiarization session before testing to avoid any learning effects. Standardized tests were conducted 48 h after hard physical training to minimize the influence of fatigue, and performed under similar conditions before and immediately after the 4-week period over 2 days. All tests were administered in the same order before and after training, and recorded by the same person. On day 1, players’ characteristics (body height and body mass), and physical performance tests were conducted in the following order: Sit and Reach, Standing Long Jump, Sprint 20-m, Illinois Agility Test, and Repeated Sprint Ability tests. The “30–15” Intermittent Fitness Test was performed on day 2. High intra-class correlation coefficients were obtained for the different performance tests varying between 0.81 and 0.98.

Height was measured on the Seca 214 portable stadiometer (Hanover, MD), body mass was measured to the nearest 0.1 kg using a digital scale (BC-554 Ironman Body Composition Monitor; Tanita, Illinois, USA), and body mass index (BMI) was calculated (BMI = body weight (kg)/body height (m^2^)).

We tested physical performance using the following tests:

Sit and Reach—a standardized sit and reach box was used for the assessment of flexibility, and the subjects were sitting with their legs stretched out in front of them and their feet flat against the vertical front end of the box. Knees must be straight. Leaning forward at the hips, the subjects needed to push the partition on the box as far as possible with their hands stretched out. The results were read out and recorded with an accuracy of 0.1 cm.

Sprint 20 m—time of a 20-m sprint in a straight line was measured by means of single beam photocell gates placed 0.3 m above the ground level (Microgate Witty, Italy). Each sprint was initiated from an individually chosen standing position, 30 cm behind the photocell gate, which started a digital timer. Each player performed three maximal 20 m sprints interspersed with 3 min of passive recovery, and the fastest time achieved was retained.

Standing Long Jump—the participant stood behind the starting line and was instructed to push off vigorously and jump as far as possible. The participant had to land with their feet together and to stay upright. Jump distance was measured from the take-off line to the back of the heel. After two familiarization tests, participants performed three repetitions. The best score of the three repetitions was selected for the analysis.

Illinois Agility Test—the length of the zone was 10 m, while the width (distance between the start and finish points) was 5 m. Four cones were placed in the center of the testing area at a distance of 3.3 m from one another. The participants started the test lying face down, with their hands at shoulder level. The trial started on the “go” command, and the participants began to run as fast as possible. The trial was completed when the players crossed the finish line without having knocked any cones over. Time was measured using a photocell system (Witty). Each player performed three trials with the best score (time) used for analysis [16].

Repeated sprint ability (RSA) test—a RSA test was used to assess repeated sprint ability [17,18,19]. The subjects ran six sprints of 40 m changing a direction of movement by 180° after 20 m. The rest between each sprint i.e., the recovery period between six sprints, was 20 s. The time was recorded with an accuracy of 0.01 s. The following variables were derived for analysis and interpretation:

RSA best time—the best sprint running time out of six sprint runs;

RSA total time—the sum of running times of all six sprint runs;

FI (fatigue index)—fatigue index was calculated according to the following formula, where *TT* is the sum of running times of all six sprint runs; *PT* is the best sprint running time out of six sprint runs; and *N* is total number of sprints completed.

(1) $$FI\left(\%\right)=\left(\frac{TT}{PT*N}-1\right)*100$$

“30–15” Intermittent Fitness Test—testing was performed in groups of up to 10 subjects. The subjects were asked to complete a run at each level of protocol given following the audio protocol [20]. Each level included 30 s of running and 15 s of active recovery. The initial or the first level included a running speed of 8.5 km/h and running speed at each subsequent level was increased by 0.5 km/h. The subjects had to reach the next zone within 30 s of running, at a given pace, with a 3 m tolerance zone. The test was interrupted as soon as the subjects failed to reach the next zone or when they gave up arbitrarily. The test results were recorded as the last successfully completed level, and the level i.e., speed reached (VIFT), was recorded. Based on the VIFT, the maximum oxygen consumption i.e., VO_2_max, was calculated according to the following formula: where *VIFT* is final running speed, *G* gender (male = 1; female = 2), *A* age, and *W* body weight (kg).

(2) $$VO_{2max\left(ml^{-1}min.kg^{-1}\right)}=28.3-2.15\;G-0.741\;A-0.0357\;W+0.0586\;A\;VIFT+1.03\;VIFT$$

### 2.3. Training Program

Both teams participated in the same weekly soccer training volume and methodology (three sessions/week of ≈90 min and one match/week). They were both visited at least once a week by our study assistants in order to monitor whether the groups used the same soccer training and if the control group did not use a structured injury prevention program but a regular warm-up. Additionally, players from both groups indicated their Rated Perceived Exertion (RPE) using the Borg’s CR-10 scale at the end of the sessions [21]. A detailed description of the usual soccer training applied during this period in both groups is depicted in Table 1.

The FIFA 11+ warm-up program was performed in three parts with a total duration of 20 min with a precisely programmed exercise load as well as precisely scheduled rests between sets. The first part involved warming up through running with performing tasks; the second part included various strength development exercises, plyometrics and stabilization, and balance exercises, and the third part also covered running including specific tasks. The program according to the instructions contained in the FIFA 11+ manual comprises three levels of difficulty depending on the athlete’s age and level of preparedness. In this research, the experimental treatment followed the beginner level. For detailed instructions for the FIFA 11+ warm-up program the manual can be found on the official website (www.f-marc.com/11 plus). The CG performed their usual warm-up consisting of a combination of running exercises (4–5 min at light intensity), followed by 4–5 min of dynamic mobility emphasizing the lower-extremity muscle groups and technical exercises with the soccer ball (4–5 min).

### 2.4. Data Analysis

The data obtained in this research were analyzed using SPSS 20.0 (IBM, Armonk, NY, USA), and are presented as mean and standard deviations. The Shapiro–Wilk test verified the normality assumption. To evaluate the effects of the FIFA 11+ program on the physical performance tests, we used a mixed ANOVA (one between-subject factor: FIFA 11+ vs. CG; one within-subject factor: pre- and post-tests). The main effects of the group and time, and their interaction effect are reported. When the main effect of time/group was significant, the simple main effect of time/group was examined. The percentage of change from pre- to post-test was also calculated for each group, and, partial eta squared (${ŋ}_{p}^{2}$) is reported as a measure of effect size. The level of significance was set at *p* ≤ 0.05.

## 3. Results

In the present study, no severe injuries were observed that would influence participation in the study. Additionally, there were no significant differences between the FIFA 11+ (5.7) and CG (5.9) for RPE, *p* > 0.05.

A mixed ANOVA showed that the mean performance of study outcomes was similar in the FIFA 11+ and CG (Table 2). However, after 4 weeks, the FIFA 11+ performance in the standing long jump was notably increased (5.6%) compared to the CG performance which decreased by −1.9%. Additionally, time spent to finish the Illinois agility test was significantly more reduced in the FIFA 11+ (−1.9%) than in CG (0.03%) after 4 weeks. The group × time interaction effect was only significant on those two tests, the standing long jump and the Illinois agility test (Table 2).

Furthermore, simple main effect of time revealed that both groups improved performance in the Sit and Reach test, the FIFA 11+ (F_(1,18)_ = 23.81, *p* < 0.001, ${ŋ}_{p}^{2}$ = 0.569) and CG (F_(1,15)_ = 7.21, *p* = 0.017, ${ŋ}_{p}^{2}$ = 0.325). Although the established level of significance (*p* ≤ 0.05) was not achieved for the interaction effect on VO_2_max, simple main effect of time showed that the mean VO_2_max significantly improved from pre- to post-test in the FIFA 11+ (F_(1,18_) *=* 22.22, *p* < 0.001, ${ŋ}_{p}^{2\;}$= 0.529), but not in the CG (F_(1,15)_ = 2.85, *p* = 0.112, ${ŋ}_{p}^{2}$ = 0.160). The changes from pre- to post-test in the remaining studied physical performance outcomes for FIFA 11+ and CG were not significant (Table 2).

## 4. Discussion

Despite the great success of the FIFA 11+ Injury Prevention Program, it was difficult, in practical terms, to persuade coaches and players to apply this program regularly solely to prevent injuries, so the aim was to prove that it also has a positive and direct impact on players’ performance. The findings of the current study indicate that the training stimuli provided by the implementation of FIFA 11+ two times per week for 4 weeks appear to be sufficient to elicit significant improvements in some (Illinois test, sit and reach, and standing long jump) but not all (20 m sprint time, RSA parameters, and VO_2_max) measures of the physical performance parameters analyzed.

The latest review revealed that the FIFA 11 s were the most commonly used prevention programs with youth soccer players [22]. Although FIFA have developed a newly constructed injury prevention program specifically tailored to younger children (FIFA 11+ kids), there is a lack of information about the effectiveness of FIFA 11+ program in youth players. 

Studies dealing with the effects of the FIFA 11+ in football have reported improvements in agility [10], balance [7,23], jump height [10], and muscle strength [7,8]. However, the aforementioned studies involved only older adolescent athletes. Only one study investigated the physical performance effects of the FIFA 11 + program in U12 soccer players [24]. The authors found a better improvement in trunk muscle endurance and similar improvements in agility times compared to a standard dynamic warm-up. Impellizzeri et al. and Zarei et al. showed that the FIFA 11+ exercises were not able to improve the results for Illinois agility test [7,11]. On the contrary, our results showed small (1.9%) but significant improvement (*p* = 0.046) for Illinois agility test after only 4 weeks of the FIFA 11+ program [7]. Similar improvements after 4 weeks also have been reported in agility run after the implementation of FIFA 11+ kids in a large cohort of football players the same age as in our study [15].

The reasons could be found in the fact that the FIFA 11+ program includes sprinting, agility, and plyometric exercises beside neuromuscular exercises [7]. Moreover, the focus in the FIFA 11+ program is on correct techniques throughout jumping, landing, and cutting movements by giving feedback about improper movement patterns [25]. The improvement may be partially due to the rise in muscle temperature [26]. Our findings are in line with programs which develop movement competency in young children, which is essential for the youth physical development model [27].

Studies looking at the influence of the FIFA 11+ in adult males have reported improvements in jump performance [28]. Positive effects on jump performance in the group of young children were found following FIFA 11+ kids [15]. Recently, Akbari et al. [12] examined the impact of the "FIFA 11+" program on the vertical jump of football players aged 17 years. Significant differences were found in favor of FIFA 11+group, but 1 month after the treatment when they stopped applying the program, the vertical jump results were back at the level of the initial measurement, which led to the conclusion that the program should be further applied since the program did not show the presence of long-term effects. Pomares-Noguera et al. showed that only 4 weeks of the application of the FIFA 11+ kids program is enough to provide better physical performance in relation to the standard method of warming up in youth football players [15]. This was confirmed in the present study where FIFA 11+ elicited significant improvement for standing long jump (5.6%; *p* = 0.001).

Different warm up routines are commonly used to optimize football performance and prevent injuries with manipulation in the content (general–specific), duration, and intensity [29,30]. According to Bishop, the longer warm-up duration is necessary to elevate baseline VO_2_, while a moderate intensity warm-up lasting 3–5 min is enough to significantly improve short-term performance [31]. The current study found that the FIFA 11+ injury prevention program only elicited positive training effects (3.08%) in comparison with the control group (1.73%), after being implemented for 4 weeks, on VO_2_max. 

The limitations of the study are the short time available to investigate the effects of the FIFA 11+ program (4 weeks), and the absence of follow-up. A follow-up assessment of the PF could have addressed the FIFA 11+ warm-up program effects on retention of PF. The generalization of the results onto girls is also a limitation because the study only included boys, thus, it is not known whether it is applicable in girls. Moreover, future studies should investigate the effects of the FIFA 11+ program with a longer duration on several physical performance variables using randomized control trial designs. Additionally, although groups did not differ in study outcomes, a lack of randomization could have contributed to bias effects, therefore, future studies should randomize participants to overcome bias.

However, the strength of the present study is that it demonstrated the plausible effects and suitability of the FIFA 11+ warm-up program even in boys aged less than 14 years. Thus, the FIFA 11+ warm-up program can be implemented in the training sessions in boys younger than 14 years old. This study supports previous findings that traditional warm-up programs could be replaced with FIFA 11+ in male youth soccer players based on its superior effects on physical performance. Moreover, this warm-up program does not require specific equipment, supports the performance development of young football players, and accordingly significantly contributes to the reduction in the injury risk. 

## 5. Conclusions

The main findings of this study suggest that just 4 weeks of implementation of the FIFA 11+ warm-up program improves physical performance compared with traditional warm-up routines in young soccer players. Therefore, the FIFA 11 + program can be considered appropriate for this age, as it seems to be adequate for inducing significant performance enhancements in young soccer players. Moreover, given the improvements in jump performance and agility, our study would advocate the introduction of these essential movement competency skills in children aged 10–12 years. Ultimately, due to evidence-based health benefits of the FIFA 11+ warm-up program, its primary target population could be expanded to recreational athletes and children in a school environment, while aiming to improve public health.

## Figures and Tables

**Table 1:** Usual soccer training session of young soccer players during intervention.

Exercise	Duration (min)
Technical drills (ball control, ball pass, ball conduction and dribbling, ball kicking, ball heading)	15
Tactical drills (defensive drills, offensive drills, situations)	20
Small-sided games with or without goal keeper and with or without change of soccer rules	20
Simulated competitive games	15

**Table 2:** Differences between Fédération Internationale de Football Association (FIFA) 11+ (n = 19) and CG (n = 17) in physical performance measures from pre- to post-test.

		Pre-Test	Post-Test	Mixed ANOVA Outcome
Sit and Reach (cm)				
	FIFA 11+	24.56 ± 5.94	26.9 ± 5.43 *	Group: F_(1, 103)_ = 0.013, *p* = 0.91, ${ŋ}_{p}^{2}$ = 0.00 Time: F_(1, 103)_ = 28.27, *p* < 0.001, ${ŋ}_{p}^{2}$ = 0.461 Group × Time: F_(1, 103)_ = 2.61, *p* = 0.115, ${ŋ}_{p\;}^{2}$ = 0.073
	CG	25.25 ± 5.91	26.43 ± 6.21 *	
Standing long jump (cm)				
	FIFA 11+	144.84 ± 18.57	153.02 ± 18.73 *	Group: F_(1, 103)_ = 1.29, *p* = 0.264, ${ŋ}_{p}^{2}$ = 0.038 Time: F_(1, 103)_ = 45.88, *p* < 0.001, ${ŋ}_{p}^{2}$ = 0.582 Group × Time: F_(1, 103)_ = 22.55, *p* < 0.001, ${ŋ}_{p}^{2}$ = 0.406
	CG	149.2 ± 14.39	152.17 ± 16.53	
Speed 20 m (s)				
	FIFA 11+	3.99 ± 0.23	4.16 ± 0.27	Group: F_(1, 103)_ = 1.6, *p* = 0.216, ${ŋ}_{p}^{2}$ = 0.046 Time: F_(1, 103)_ = 0.46, *p* = 0.505, ${ŋ}_{p}^{2}$ = 0.014 Group × Time: F_(1, 103)_ = 3.68, *p* = 0.064, ${ŋ}_{p}^{2}$ = 0.1
	CG	3.89 ± 0.37	3.97 ± 0.33	
Illinois Agility Test (s)				
	FIFA 11+	18.63 ± 0.93	18.27 ± 0.93 *	Group: F_(1, 103)_ = 3.68, *p* = 0.126, ${ŋ}_{p}^{2}$ = 0.07 Time: F_(1, 103)_ = 6.9, *p* = 0.013, ${ŋ}_{p\;}^{2}$ = 0.173 Group × Time: F_(1, 103)_=7.91, *p* = 0.008, ${ŋ}_{p}^{2}$ = 0.193 F_(1, 103)_ = 7.91, *p* = 0.008, ${ŋ}_{p}^{2}$ = 0.193
	CG	18.04 ± 1.21	18.05 ± 1.19	
RSA BT (s)				
	FIFA 11+	9.58 ± 0.71	9.47 ± 0.69	Group: F_(1, 103)_ = 1.74, *p* = 0.197, ${ŋ}_{p}^{2}$ = 0.050 Time: F_(1, 103)_ = 2.78, *p* = 0.105, ${ŋ}_{p}^{2}$ = 0.078 Group × Time: F_(1, 103)_ = 0.061, *p* = 0.807, ${ŋ}_{p}^{2}$ = 0.002
	CG	9.5 ± 0.60	9.43 ± 0.6	
RSA TT (s)				
	FIFA 11+	60.63 ± 3.98	61.66 ± 8.2	Group: F_(1, 103)_ = 1.64, *p* = 0.21, ${ŋ}_{p}^{2}$ = 0.047 Time: F_(1, 103)_ = 1.124, *p* = 0.297, ${ŋ}_{p}^{2}$ = 0.033 Group × Time: F_(1, 103)_ = 0.36, *p* = 0.551, ${ŋ}_{p}^{2}$ = 0.011
	CG	58.86 ± 3.4	59.29 ± 3.4	
Fatigue index (%)				
	FIFA 11+	5.69 ± 2.91	5.92 ± 2.66	Group: F_(1, 103)_ = 0.126, *p* = 0.725, ${ŋ}_{p}^{2}$ = 0.004 Time: F_(1, 103)_ = 0.01, *p* = 0.916, ${ŋ}_{p\;}^{2}$ = 0.00 Group × Time: F_(1, 103)_ = 0.001, *p* = 0.976, ${ŋ}_{p}^{2}$ = 0.00
	CG	6.18 ± 2.45	6.23 ± 2.45	
VO_2_max (mL/kg/min)				
	FIFA 11+	39.52 ± 2.45	40.74 ± 2.45*	Group: F_(1, 103)_ = 0.3, *p* = 0.865, ${ŋ}_{p}^{2}$ = 0.001 Time: F_(1, 103)_ = 18.32, *p* < 0.001, ${ŋ}_{p}^{2}$ = 0.357 Group × Time: F_(1, 103)_ = 3.2, *p* = 0.083, ${ŋ}_{p}^{2}$ = 0.088
	CG	40.25 ± 3.31	40.95 ± 3.37	

Values are mean±SD. Abbreviations: F—F test value; *p*—statistical significance; ${ŋ}_{p}^{2}$—partial eta squared; RSA BT—best sprint running time out of six sprints; RSA TT—total time of all six sprints; * significant pre-to post-test change.
